# Yoda1, a Piezo1 agonist, induced latent HIV reactivation associated with upregulation of CD3/TCR complex and HLA genes

**DOI:** 10.21203/rs.3.rs-6208371/v1

**Published:** 2025-04-10

**Authors:** Alexander Bontempo, Alireza Heidari, Maria Rita Pastore, Riccardo Madonia, Adam Sadik, Mark Schweizer, Mark Cayabyab

**Affiliations:** Nova Southeastern University College of Dental Medicine; Nova Southeastern University College of Dental Medicine; Nova Southeastern University College of Dental Medicine; Nova Southeastern University College of Dental Medicine; Nova Southeastern University; Nova Southeastern University College of Dental Medicine; Nova Southeastern University College of Dental Medicine

**Keywords:** Yoda1, Piezo1, HIV latency, LRA, omics

## Abstract

There is currently no cure for HIV because of the presence of latent viral reservoirs in people with HIV (PWH) on antiretroviral therapy (ART). Latency-reversing agents (LRAs) that can effectively reactivate and destroy latent HIV are being developed as a possible cure for HIV. Here, we identify Yoda1, a Piezo1 agonist, as a novel LRA. Yoda1 reactivated latent HIV in vitro ACH2 cells and ex vivo PBMCs from an HIV patient on ART. Yoda1 induced infectious virus production and HIV gene expression via Piezo1 activation and calcium signaling. Transcriptomic and proteomic analyses revealed a unique latent HIV reactivation pathway involving T cell activation, upregulation of TCR/CD3 and HLA genes, as well as modulation of host and viral transcription and translation that favors viral gene expression. These findings suggest further testing and development of Yoda1 as an effective LRA to reactivate latent HIV and destroy latent reservoirs for the cure of HIV.

## INTRODUCTION

Over the course of its four-decade pandemic trajectory, HIV has resulted in approximately 41 million deaths. In 2023, 40 million people were living with HIV, 630,000 individuals have died from HIV-related causes, including 76,000 children under the age of 15.^1^ HIV antiretroviral therapy (ART) stands as one of the most remarkable achievements in contemporary medicine. Before the advent of ART, the mortality rate among individuals infected with HIV was nearly 100%.^2^ Currently, people living with HIV (PWH) on ART who maintain controlled viremia can expect a life expectancy and quality of life comparable to that of the general population.^2^

Despite the extraordinary benefits of ART, HIV remains a chronic condition, and patients must rely on ART to sustain their health. Unfortunately, access and adherence to ART often present significant challenges, limiting the effectiveness of treatment regimens. Financial barriers and psychological factors, often exacerbated by the stigma surrounding HIV, are correlated with reduced ART success rates.^3–5^ The United States’ contributions and response organizations like PEPFAR have saved more than 26 million lives through treatment, care, and support. Potential clinical and economic impacts of cutbacks in the President’s Emergency Plan for AIDS Relief Program globally will be catastrophic, leading to 565,000 new infections, 100,000 new deaths, and the loss of 3.71 life years in South Africa alone.^6,7^

The development of a vaccine, as well as a sterilizing or functional cure, remains crucial for the ultimate eradication of HIV. From the global perspective, the need for a cure becomes even more critical. There has been a dedicated research effort to develop treatment strategies to clear the virus from infected individuals and PWH on suppressive ART, but the existence of a latent population of cells harboring integrated HIV provirus has proven difficult to target. Memory CD4 T cells comprise most of the HIV reservoir, but HIV is also detected in various cell types and anatomical locations, including the lymph nodes, gastrointestinal tract, central nervous system, lungs, bone marrow, and genital tract.^8,9^ In the bloodstream, myeloid cells such as monocytes, macrophages, and follicular dendritic cells also harbor HIV.^10,11^ Compounding the difficulty of detecting and quantifying the reservoir, it is estimated that only one CD4 T cell per million contains HIV DNA, much of which is non-infectious and consists of incomplete viral genomes.^12–14^

The HIV-infected CD4 T cell reservoir is highly diverse, harboring transcriptionally quiescent provirus that persists during ART and sustained by homeostatic proliferation and survival.^15^ When ART is discontinued, the virus reactivates and rebounds to re-establish a propagating infection.^16^ Viral reservoirs exhibit persistent viral replication kinetics and are highly resistant to ART, as no current ART agents are specifically designed to target HIV gene expression and replication. Viral reservoirs play a critical role in re-establishing active infection shortly after ART cessation, even after prolonged periods of uninterrupted treatment. It is now widely accepted that the reactivation of latent HIV within the reservoir is a pivotal step towards achieving a definitive cure. Upon reactivation, infected cells can be rapidly eliminated through direct viral cytopathic effects and immune-mediated responses.

There are ongoing research efforts to develop HIV cure strategies using the “kick-and-kill” approach.^17^ This two-pronged approach involves the reactivation of latent HIV-infected cells for the “kick”^10,18^ using a class of drugs called Latency Reversal Agents (LRA). These are designed to reactivate latent HIV-infected cells to flush out the virus and render the virus and these cells susceptible to the “kill” by ART and immunologic clearance. Current LRAs interfere with chromatin remodeling and gene silencing, which were shown to be involved in HIV latency. Key targets in this context include DNA methyltransferases (DNMTs), histone deacetylases (HDACs), and histone methyltransferases (HMTs).^19–22^ Several inhibitors and agonists acting on chromatin remodeling have shown the ability to induce HIV expression in latently infected cells.

Many studies have examined strategies to reverse HIV latency by inducing virus reactivation and viral antigen re-expression to restore immune detection of infected cells.^20^ However, LRAs studied and clinically tested to date have failed due to inefficient HIV reactivation, highlighting a knowledge gap in HIV latency reversal.^19,21,23^ Indeed, studies in PWH show that, on average, only 1.7% of intact proviruses across all T cell subsets were induced in vitro to transcribe viral genes and release replication-competent virus after stimulation.^24^ Hence, there is an urgent need for a better understanding of the mechanisms involved in effective latency reversal and the development of more effective and safer LRAs for HIV cure.

In this study, we propose the Piezo1 agonist, Yoda1, as a novel and effective LRA in a kick-and-kill strategy. Described herein, we provide strong functional data supporting the ability of Yoda1 to reactivate latent HIV.

## MATERIALS AND METHODS

### Cells

ACH-2 cells from AIDS Reagent Program were cultured in RPMI 1640 (Gibco, Waltham, MA) with 10% Fetal Bovine Serum, 10mM HEPES, at 37°C and 5% CO_2_. The TZM.bl cells from AIDS Reagent Program were cultured in DMEM (Gibco, Waltham, MA) with 2% PEN-STREP (Gibco, Waltham, MA) and 10% Fetal Bovine Serum at 37°C and 5% CO_2_.

### Piezo1 activation

For the chemical activation of the Piezo1 calcium channel, 5 × 10^5^ ACH2 cells per ml were treated with 15μM Yoda1 (Sigma Aldrich, St. Louis, MO) at 37°C for 72 hours. Activated cells were centrifuged at 2500 RPM for 5 minutes and supernatant was collected. The supernatant was used to infect TZM.bl to assess virus production.

### Cell viability assay

The viable cells were counted in 1:1 cell suspension and 0.4% solution Trypan Blue (Gibco, Waltham, MA) mix using Invitrogen Countess 3 Automated Cell Count (Gibco, Waltham, MA) in triplicates.

### TZM.bl infection assay

The TZM.bl cells were washed with 5ml PBS pH 7.2 (without Calcium Chloride and Magnesium Chloride) (Gibco, Waltham, MA) and trypsinized with 2ml Trypsin for 15 minutes. 2 × 10^4^ cells were seeded and adhered to 96 Cell Culture Plates (SPL Life, Gyeonngi-do, South Korea) at 37°C and 5% CO_2_. The TZM.bl cells were then infected with 100μl of the ACH-2 supernatant for 72 hours to assess viral production.

### LTR transactivation assay

pALPS Tat-P2A-Rev plasmid (Addgene, Watertown, MA; Cat#101331) transfection was carried out following Lipofectamine 2000 standard protocol. Briefly, 6-well plate wells with 80% confluent 293T cells, culture medium was replaced with 2ml OptiMEM. 250 μl OptiMEM containing 10μl Lipofectamine 2000 and 250μl OptiMEM with 4μg plasmid were incubated at RT for 5 minutes. The solutions were mixed, incubated for 15 minutes at RT and subsequently added to the cell culture dropwise, while swirling the plate to allow optimal dilution. On the following day, the medium was replaced with DMEM/10% FBS. Cells were incubated 37°C and 5% CO2 overnight and then used for downstream Yoda1 transactivation assay.

### Lentivirus production and infection assay

5 × 10^6^ 293T cells in T75 flask containing a 1:1 mixture of optiMEM (GIBCO, Waltham, MA) and DMEM supplemented with 10% FBS (GIBCO, Waltham, MA) were transfected as follows: 14.2μg pHAGE-Luc-zGFP (BEI Resources, Manassas, VA), 16μg pCMVdR8.2 (Addgene, Watertown, MA), and alternatively 6.4μg pcDNA3.1-BG505 (kindly provided by Dr. John P. Moore, Cornell University, Ithaca, NY) or pcDNA3.1-ZM197M.PB7 (NIH HIV reagent Program, Bethesda, MD) were dissolved in 1.8ml optiMEM. 1.8ml of optiMEM with 75μl of Lipofectamine 2000 (Invitrogen, Waltham, MA) was then added to the plasmid mixture after incubation at RT for 5 minutes. The mix was incubated at RT for another 15 minutes. The final solution was added dropwise onto the cells. The day after transfection, the medium was replaced with 15ml DMEM/10%BSF/2% PEN-STREP with 1x non-essential amino acids (GIBCO, Waltham, MA). Supernatants were collected after 24 and 48 hours. Supernatants were filtered through a 0.45 μm PVDF Millex-GV filter (Millipore, Burlington, MA). Lenti Concentrator reagent (OrigeneOrigen, Rockville, MD) at 1:5 ratio was added to the filtered medium and incubated for 2–4 hours at 4°C. The mixture was centrifuged at 4°C for 40 minutes at 4,000 RPM, and the viral pellet was resuspended in DMEM to a 1/75th of the initial volume. The virus was stored at −80°C before use.

Virus infection was performed as follows: 5,000 TZM.bl cells per well in 96-well plate in complete DMEM growth medium containing 15μM Yoda1 were infected with 10μl virus solution for 48 hours. Infection was assessed using a luciferase assay described below.

### Luciferase assay

To assess virus infection, luciferase expression was measured using a Synergy H1 Plate Raeder (BioTek, Winooski, VT). The medium in the 96 well plate was discarded and 180μl of a solution 1:1 of BriteLite Plus (Perkin-Elmer, Waltham, MA) and complete RPMI1640 was added. Cells were lysed by repeated pipetting and 150 μl of the cell lysate was transferred into a white 96-well plate for luminescence measurement.

### Cell membrane and nuclear fluorescent staining

Infected TZM.bl cells were washed with PBS and incubated with 1X Cellbrite 555 (Biotium, Fremont, CA) in PBS for 15 minutes. The cells were then washed with PBS and fixed using 4% PFA in PBS for 10 minutes and washed again. A solution of DAPI in PBS was added and cells were visualized using a LSM540 confocal microscopy (Zeiss, Oberkochen, Germany).

### Gene expression analysis by RT-qPCR

Yoda1-treated ACH-2 cells were pelleted by centrifugation at 2500RPM for 5 minutes. Total RNA was extracted using Qiazol Lysis reagent (Qiagene, Venlo, the Netherlands). Total RNA was quantified by nanodrop and 28–100ng were used for cDNA synthesis. cDNA for Gag quantification was produced using Gag and GAPDH reverse primers to assure cDNA specificity while for Piezo1 a 1:5 mixture of random hexamers and Poly-T was used. cDNA was obtained using VERSO cDNA kit (Thermo Fisher Scientific, Waltham, MA) following manufacturer instruction. cDNA synthesis was performed at 42°C for 40 minutes followed by 2 minutes at 95°C. 2μl of cDNA were used for PCR quantification using Quantabio perfecta SYBR Green superMix low ROS (Quantabio, Beverly, MA) in QuantStudio 3 PCR device (Applied Biosystems, Waltham, MA). Primers used for detection of Gag and GAPDH used at 400nM final concentration were the following: Gag Forward: CCACCTATCCCAGTAGGAG; Gag Reverse: CTCCCTGACATGCTGTCATC; GAPDH Forward: TCAAGGCTGAGAACGGGAAG; GAPDH Rev: CGCCCCACTTGATTTTGGAG.

TaqMan assay to detect the expression of Piezo1 in ACH2 cells was performed using TaqMan Fast Advanced Master Mix (Thermo Fisher Scientific, Waltham, MA). In brief, 10μl of master mix were added to 1μl of Piezo1 TaqMan Assay kit (Thermo Fisher Scientific, Waltham, MA; Cat# Hs00207230_m1) containing specific primers and probe and 1μl of cDNA in a 20μl final volume with milliQ water. PCR was performed in an AB QuantStudio 3 equipment (Applied Biosystems, Foster City, CA) using the built-in fast protocol as thermocycling profile. The threshold value was registered and a value less than 30 was considered positive for Piezo1 expression.

### Yoda1 activation of PWH PBMC

De-identified peripheral blood mononuclear cells (PBMC) were isolated from a PWH patient with Nova Southeastern University Institutional Review Board (IRB) approval. Two Histopaque 1077 (Sigma Aldrich, St. Louis, MO) PBMC separation aliquots were processed following manufacturer’s protocol. Briefly, 4ml of Histopaque 1077 solution was aliquoted in two 15ml conical tubes. In each tube, 4ml blood was carefully layered and centrifuged for 30 minutes at 4000xg at RT. After centrifugation, the interphase containing the cell was isolated. The two cell aliquots were pooled and washed twice with 10ml PBS and resuspended in 6.4ml RPMI1640/10% FBS. The cell suspension was divided into 3 wells in a 6-well plate containing 2×10^6^ cells each well. One well was treated with 15μM Yoda1 and another with 5ng/ml of PMA. After 72 hours, the cells were counted and viability assessed by trypan blue staining. The presence of virus was determined using TZM.bl infection assay as described above. Data were analyzed by one-way ANOVA and Tukey’s post-hoc test.

### Proteomic analysis

Proteins were reconstituted in a buffer containing 2% (w/v) SDS, 50mM Tris pH 8.0. Subsequently, 100μl protein extract was treated with 10μl of 250mM TCEP at 70°C for 10 minutes. This step was followed by incubation with 20μl of 400mM IAA at room temperature for 10 minutes. Purification and tryptic digestion of proteins were performed using the SP3 protocol, as outlined by the original authors.^25^ The resulting peptides underwent purification using Stage Tips with SDB-RPS sorbent, following the protocol developed by *Rappsilber et al*.^26^

The Vanquish Neo nanoLC system, Orbitrap Eclipse mass spectrometer, FAIMS Pro Interface, and Easy Spray ESI source (Thermo Fisher Scientific, Waltham, MA), were used for LC-MS/MS data measurements. NanoLC separation was performed using a combination of an Acclaim PepMap trap column (75μm × 2cm) and an EasySpray ES802 column (75μm × 25mm, 100Å) (Thermo Fisher Scientific, Waltham, MA). 5μl peptide digest was injected, and the analytes were separated with a mobile phase comprising 0.1% (v/v) formic acid in water (solution A) and 0.1% (v/v) formic acid in 80% (v/v) acetonitrile (solution B), flow rate at 300nL/min and the column temperature at 40°C. Initial equilibration of the column with 3% solution B for 2 minutes was followed by a gradual linear gradient (raised to 40% solution B) for 60 minutes. Any residual peptides attached to the C18 resin were eluted with 95% solvent B for 11 minutes. In positive MS mode, the ion source temperature was set at 305°C, and ionized peptides were separated from singly charged ions through the FAIMS Pro unit at −50V. Mass spectra were collected in MS1 mode with a resolution of 120,000, covering the mass range of m/z 350–2000, using standard automatic gain control (AGC) settings and automatic injection time. For MS/MS fragmentation, a data-independent acquisition (DIA) mode a range of m/z 375–1200 and a 30,000 resolution was implemented. The collision energy was set at 30%, and an AGC target of 1000% was applied. MS2 spectra were measured across m/z 25 isolation windows with 0.5 m/z overlaps.

Proteome data analysis was carried out utilizing the DIA-NN 1.8.1 software platform.^27^ LC-MS data files underwent processing and analysis with the following parameters: FASTA database – Homo sapiens (UP000005640) and HIV (UP000007692); FASTA digest for library-free search/library generation; Protease – Trypsin/P missed cleavages – 1, N-term M excision; C carbamidomethylation; Peptide length range – 7–30; Precursor charge range – 1–4; precursor m/z range – 300–1800; fragment ion m/z range – 200–1800; Precursor FDR (%) – 1.0; Use isotopologues; heuristic protein interference; no shared spectra; Protein interference – Genes; Neural network classifier – Single-pass mode; Quantitation strategy – Robust LC (high precision); Cross-run normalization – RT-dependents; Library generation – Smart profiling; Speed and RAM usage – optimized for optimal results. Raw protein peak areas were utilized for statistical analysis, following the methodology outlined by *Schulte et al*.^28^

### Transcriptomic analysis

Total RNA was extracted from fresh frozen cell pellets using the Qiagen RNeasy Plus Mini kit following manufacturer’s protocol (Qiagen, Hilden, Germany). RNA samples were quantified using a Qubit 2.0 Fluorometer (Life Technologies, Carlsbad, CA). RNA integrity was evaluated using an Agilent TapeStation 4200 (Agilent Technologies, Palo Alto, CA). RNA sequencing libraries were prepared using the NEBNext Ultra II RNA Library Prep Kit for Illumina (NEB, Ipswich, MA). Brie y, enriched mRNAs using Oligo(dT) beads were fragmented for 15 minutes at 94°C. First-strand and second strand cDNA were synthesized. The cDNA fragments were end-repaired and adenylated at 3’ ends, and universal adapters ligated to cDNA fragments, followed by index addition and library enrichment by PCR with limited cycles. Sequencing libraries were validated on the Agilent TapeStation (Agilent Technologies, Palo Alto, CA) and quantified by using Qubit 2.0 Fluorometer (Invitrogen, Carlsbad, CA) as well as by qPCR (KAPA Biosystems, Wilmington, MA). The sequencing libraries were clustered on a flow cell ready for sequencing using Illumina NovaSeq. The samples were sequenced using a 2×150bp Paired-End (PE) configuration, targeting 30M reads/sample. The raw sequencing data (.bcl files) were converted into fastq files and de-multiplexed using Illumina’s bcl2fastq 2.20 software. One mismatch was allowed for index sequence identification. Data Analysis Sequence reads were trimmed to remove possible adapter sequences and nucleotides of poor quality using Trimmomatic v.0.36. The trimmed reads were mapped to the human reference genome (GRCh38) available on ENSEMBL using the STAR aligner v.2.5.2b. The STAR aligner is a splice aligner that detects splice junctions and incorporates them to help align the entire read sequences. Mapping .bam files were used to generate hit counts for genes and exons. Unique gene hit counts were calculated using feature CONFIDENTIAL Counts from the Subread package v.1.5.2. Unique reads within exon regions were counted and used in downstream analysis.

### Differential gene expression, clustering and pathways/ontology analyses

Proteomic and transcriptomic read counts were analyzed using Bigomics Playground platform. Statistical significance of the differential expression was expressed as meta.q, which is the most conservative analysis that integrates three different statistical methods: DESeq2 Wald, edgeR and Limma. Differential gene expression was determined using the threshold optimized to best fit the distribution of each data set, but in all cases following a strict minimal standard (Fold-Chage > 1.5 and meta.q < 0.05). The global transcriptional and proteomic changes across samples were represented by a volcano plot to determine downregulated and upregulated genes. Bigomics Playground was also used to run gene ontology and pathways analysis to determine the gene functions significantly influenced by the Yoda1 induction (i.e., biological processes, molecular functions, and cellular components).

## RESULTS

### Yoda1 induced the reactivation of latent HIV in ACH2 cells leading to the production of infectious HIV.

**Figure 1:**
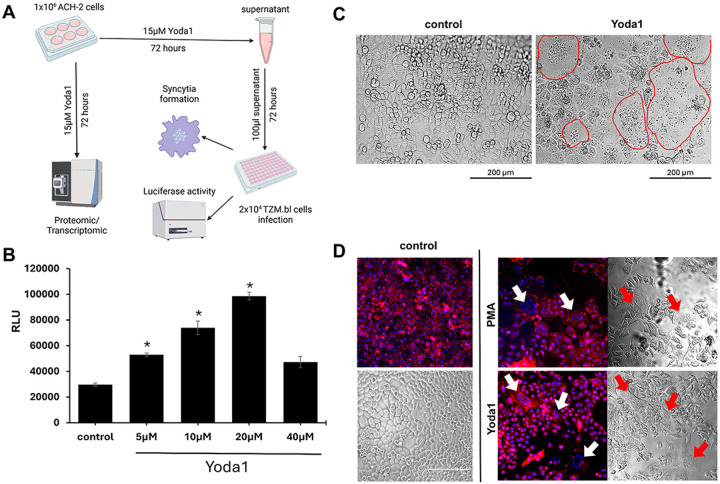
Yoda1 reactivated HIV in the vitro latent HIV model ACH2 CD4 T cell. A) Diagram describing the assessment of HIV reactivation in ACH2 cells. Yoda1 reactivates ACH2 to produce infectious HIV which is assessed by the ability of supernatants to infect TZM.bl (measured by luciferase activity) and form syncytia. B) Yoda1 dose-response curve showing maximal reactivation at 15–20μM. Statistical difference between Yoda1 treatment and control was assessed by one-way ANOVA and Tukey’s post-hoc test (*=p-value<0.05) (F_5,12_= 93.56, p=3.49×10^−9^). C) TZM.bl syncytia whereas induced by 15 μM Yoda1, visualized, with light microscopy at 20X, as giant cells (circumscribed in red) containing multiple nuclei. D) Syncytia were visualized by confocal microscopy. Nuclei were stained with DAPI (blue) and cell membranes using CellBrite 550 (red). Bright field was co-visualized as reference. Treatment with Yoda1 and PMA induced syncytia indicated by arrows.

Piezo1 is expressed in various cell types, including CD4 T cells, mediating cell activation via calpain activation and organization of cortical actin scaffold, TGFβ and integrin-dependent chemotactic migration signaling.^29–31^ We addressed whether the Piezo1 agonist, Yoda1 could reactivate latent HIV CD4 T cells using ACH2 cells. Our experimental design for assessing HIV reactivation by Yoda1 is shown (Fig. 1A). Following a 72-hour treatment with Yoda1, ACH2 produced infectious HIV capable of infecting TZM.bl cells, achieving maximum virus production and infection (measured by luciferase activity) at 15–20μM (Fig. 1B). Infection of TZM.bl cells resulted in the formation of syncytia (Fig. 1C). Fluorescence staining of the syncytia revealed a diffuse disruption of cell-cell adhesion, indicating a cytopathic effect due to infection (Fig. 1D).

### Yoda1 reactivated latent HIV in ACH2 cells, leading to the upregulation of HIV genes.

Reactivation of latent HIV was previously shown to result in the expression of HIV genes in latent cells.^20,32^ Proteomics analysis revealed that Yoda1 markedly upregulated the expression of HIV-1 Gag-Pol proteins (Fig. 3A) and HIV-1 gp160 (Fig. 3B). We also found that Yoda1 induced the HIV-1 Gag mRNA expression (Fig. 3C), and RNAseq analysis showed that Yoda1 upregulated the expression of HIV BRU RNA (Fig. 3D). Altogether, these results demonstrate that Yoda1 reactivated latent HIV by inducing the expression of HIV-1 genes.

### Piezo1 is expressed on ACH2 and mediated reactivation of latent HIV by Yoda1.

The interaction of Yoda1 with Piezo1 receptor results in the activation of T cells.^31,33^ We addressed the role of Piezo1 in the reactivation of latent HIV. First, we found that Piezo1is expressed in ACH2 cells (Fig. 3A). To assess the involvement of Piezo1, we used EGTA and streptomycin, which are inhibitors of Piezo1. EGTA sequesters calcium cations required for the Piezo1 signaling cascade while streptomycin has an activation-dependent inhibitory effect.^34^ Both Piezo1 inhibitors abrogated latent HIV reactivation with significant reduction of HIV-1 virus production and syncytium formation (Fig. 3B and C, respectively). Additionally, EGTA prevented the upregulation of HIV Gag expression after Yoda1 stimulation (Fig. 3D). Collectively, these results suggest that the Piezo1 signaling pathway plays an important role in latent HIV reactivation induced by Yoda1.

### Yoda1 reactivated latent HIV in PWH PBMC.

Most PWH under ART treatment are virally suppressed but have quiescent latent HIV infection.^8,18,32^ We found that Yoda1 treatment of human PBMC from a PWH under ART and with undetectable viral load, markedly reactivated latent HIV *in vitro* by inducing the production of infectious HIV compared to the untreated control (Fig. 4A). EGTA inhibited reactivation of latent HIV in PWH PBMC (Fig. 4A). The level of Yoda1-mediated reactivation of PWH was comparable to PMA (Fig. 4A). The effects of Yoda1 and Piezo1 inhibitors were not due to compromised viability (Fig. 4B). All these data combined suggest that Yoda1 can strongly reactivate latent HIV in PWH PBMC that is mediated by Piezo1.

### Yoda1 reactivated HIV by inducing LTR transactivation.

Upregulation of HIV gene expression during latent HIV reactivation is mediated by LTR transactivation.^35^ We assessed the role of LTR transactivation in latent HIV reactivation that is mediated by Yoda1. Using an LTR-luciferase assay, we found that treatment of TZM.bl cells containing an LTR-luciferase cassette with 15μM Yoda1 resulted in LTR transactivation (Fig. 5A). Infection of highly susceptible TZM.bl containing the LTR-luciferase cassette with pseudovirus expressing the envelope glycoproteins of either the primary isolate BG505 (Clade A) or ZM197M (Clade C) dramatically increased luciferase activity (Fig. 5b). In addition, we found that Yoda1 treatment of TZM.bl transfected with pALPS-Tat-P2A-rev increase luciferase activity by approximately 2-fold (Fig. 5C). Taken together, these results strongly suggest that Yoda1 can reactivate latent HIV by upregulating the expression HIV genes through the transactivation of the LTR.

### Proteomic analysis showed that Yoda1 upregulated T cell and immune response markers.

We conducted proteomic analysis to determine host factors that may be involved in latent HIV reactivation. A total of 6 independent proteomic replicates for each ACH2 control and ACH2 treated with 15μM Yoda1 were clustered by UMAP method. The replicates of each condition clustered well in the 2D space showing that latent cells treated with Yoda1 had a distinct proteomic profile (Fig. 6A). The statistical significance of the differential expression was calculated employing 3 methods (Wald test, EdgeR and Limma trend). Considering the average among the three methods and adopting an FDR of 0.2 or less, Yoda1 treatment of latent cells was characterized by the upregulation of a total of 411 and the downregulation of 74 proteins (Fig. 6A). In line with the differential expression analysis, the volcano plot showed a skewed distribution of upregulated proteins towards the upper-right (Fig. 6B). In Fig. 6C, the 50 most differentially expressed proteins clustered by treatment status are represented.

The differential expression analysis revealed that Yoda1 upregulates a set of genes related to immune response and T cells. Yoda1 was associated with the expression of T cell receptor (TCR) components belonging both to αβ (TRA/TRAC, TRAV29DV5, TRBV3–1) and γδ (TRGV3, TRGC1) subtypes (Fig. 7). Yoda1 affected also the CD3 co-receptor complex as shown by the upregulation of CD3D, CD3E, CD3G and CD247 (CD3Z) (Fig. 7).

**Figure 2:**
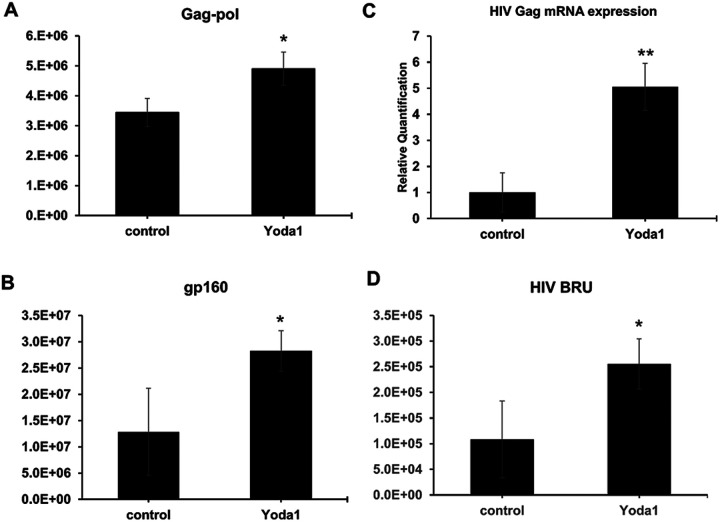
Yoda1 reactivated latent HIV in ACH2 resulting in the upregulation of HIV genes. A and B) Proteomic analysis revealed Yoda1-mediated increase in HIV Gag-pol and gp160 proteins expression. C) RT-qPCR analysis showed that Yoda1 increased Gag mRNA (5-fold) relative to control. D) RNAseq showed that Yoda1 increased HIV-1 BRU (K02013.1) RNA. Statistical difference between the group means was assessed by two-tailed Student’s T test (*=p-value<0.05, **=p-value<0.01).

**Figure 3:**
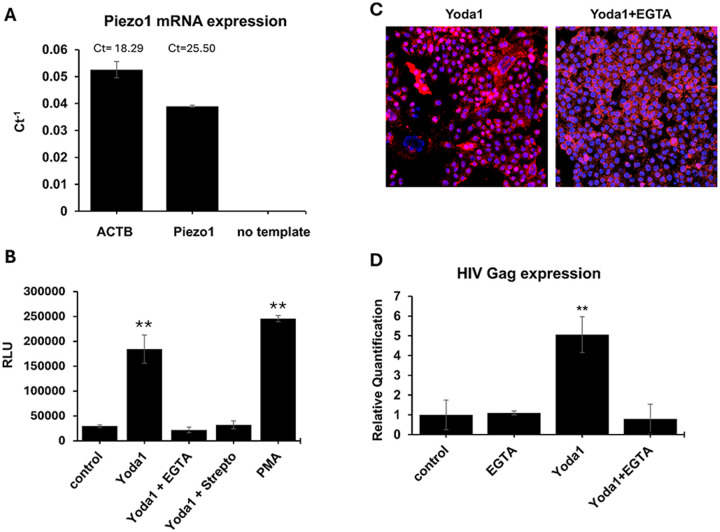
Piezo1 is expressed on ACH2 CD4 T cell clone and mediated the reactivation of latent HIV by Yoda1. A) Two independent RT-qPCR analyses showed ACH2 cells expresses Piezo1. Results indicated a consistent Piezo1 expression with a threshold cycle (Ct) of 25.50 compared to the positive control, Actin (ACTB) and negative control Piezo1 primers only (no template). ACTB was quantified as internal reference. B) Yoda1 (15 μm) co-administered with 200ug/ml streptomycin (Strep), a known Activationdependent Piezo1 inhibitor, or 1mM EGTA that specifically sequester Ca2+, inhibited latent HIV reactivation. PMA (5ng/ml) was used as positive control. Data were analyzed by one-way ANOVA and Tukey’s post-hoc test (**=p-value<0.01) (F_5,12_= 213.47, p=2.73×10^−11^). C) Using confocal microscopy, EGTA impaired the formation of syncytia indicating poor virus production in response to Yoda1 treatment. D) RT-qPCR analysis showed that EGTA inhibited HIV Gag upregulation by Yoda1. Statistical difference between the group means was assessed by two- tailed Student’s T test (**=p<0.01).

**Figure 4:**
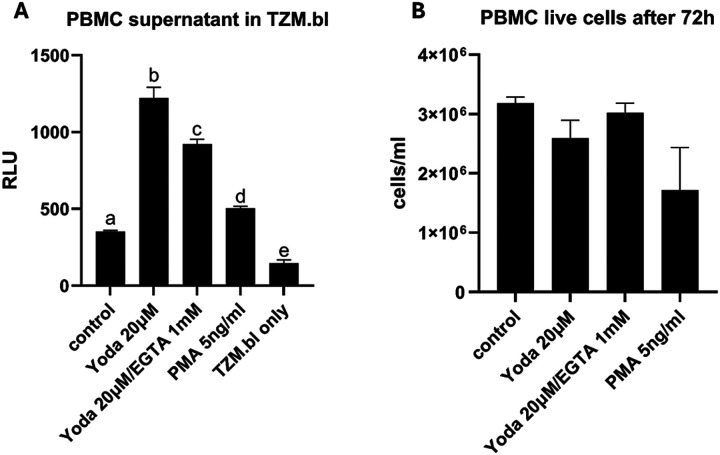
Yoda1 reactivated HIV in PWH PBMC. A) PBMCs (2×106 cells) of PWH undergoing ART with undetectable HIV was treated with 15 μM Yoda1 for 72 hours. Supernatant of the Yoda1-treated PWH PBMC produced viruses capable of infection of TZM.bl cells (measured by RLU). EGTA (1mM) reduced latent HIV reactivation. PMA (5ng/ml) reactivation was less than Yoda. Unstimulated (control) and TZM.bl only controls are shown. B) Treatment with Yoda1 for 72 hours had no significant effect on cell viability measured by trypan-blue cell staining. The mean of each group in triplicates was statistically different from each other, shown by the letters a-e (ANOVA one-way, Tukey’s post-hoc) (F_4,10_=442.2, p<0.0001).

**Figure 5:**
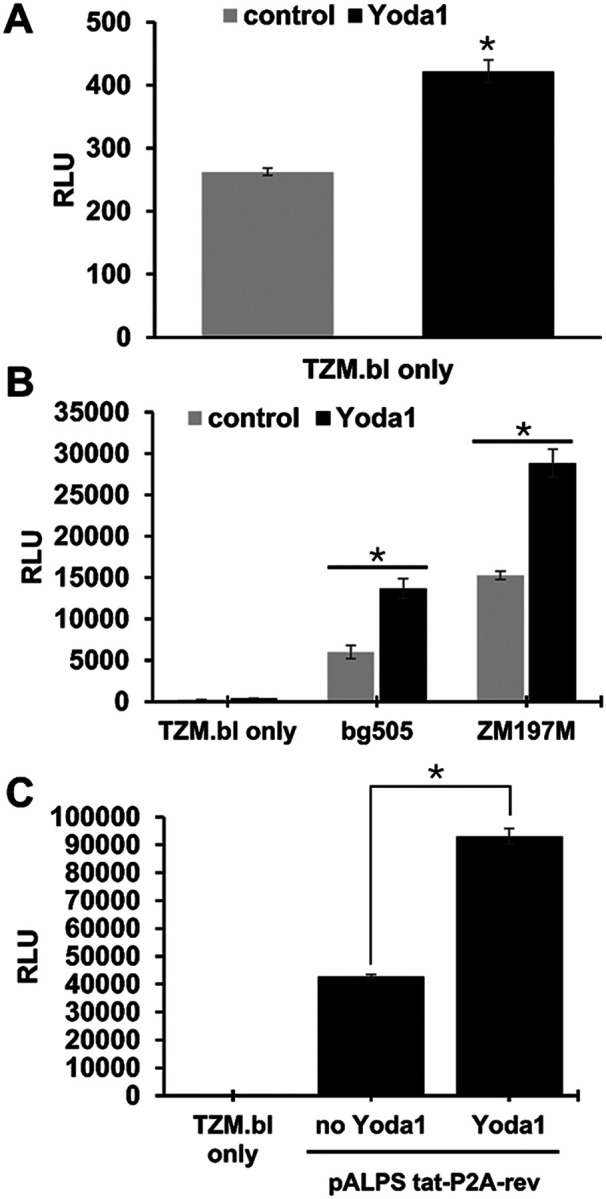
Yoda1 reactivated latent HIV by inducing LTR transactivation. A) Yoda1 (10μM) increased base line luciferase activity in TZM.bl without the HIV LTR transactivation protein, Tat. B) Yoda1 (10–15μM) significantly increased virus infection, using pseudotype lentivirus expressing either tier-2 HIV-1 BG505 or ZM197M ENV glycoproteins. C) LTR Transactivation, measured by luciferase activity, was assessed using TZM.bl containing a stable copy of an LTR-Luc expression cassette. Yoda1 had a synergistic effect on transactivation with Tat and Rev protein co-expression. Yoda1 increased transactivation by 100% compared to Tat-Rev stimulation alone (no Yoda1). Statistical difference between the group means was assessed by two-tailed Student’s T test (*=p<0.05).

**Figure 6:**
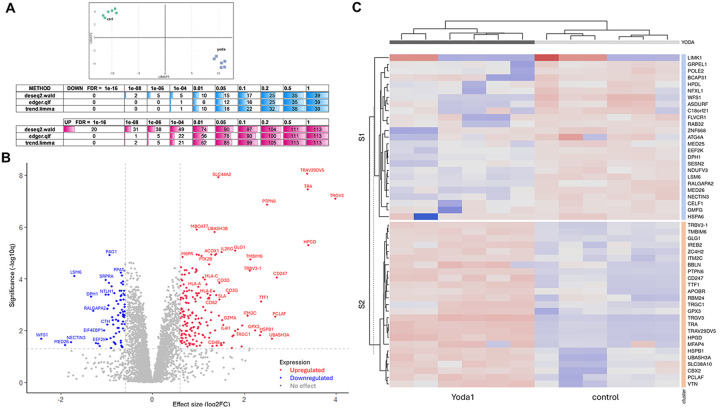
HIV reactivation by Yoda1 in ACH2 cells involveda novel signaling pathway. A) The proteomic profile of ACH2 (6 replicates) treated with 15μM Yoda1 and 6 control replicates were graphed using Uniform Manifold Approximation and Projection (UMAP). The Samples clustered well congruently to Yoda1 treatment. Upregulated (in blue) and downregulated (in red) proteins were statistically analyzed using three alternative tests: DESeq2 Wald, edgeR and Limma. Meta,q is the most conservative value calculated by the three tests. Downregulated (blue) and upregulated proteins in Yoda1 vs. control samples were grouped by False Discovery Rate (FDR) p-value range. B) Volcano plot is a simple graphical representation of the protein differential expression between ACH2 treated with 15μM Yoda1 versus control cells. Red and blue dots represent upregulated and downregulated proteins, respectively. Cut-off for Fold-Change (FC) was set to 1.5. Statistical significance cut-off was set to 0.05. Effect size is represented by logarithm base two of the FC while significance was plotted as the negative logarithm base ten of the Meta.q value for each protein. C) The 50 most differentially expressed proteins were clustered. The upregulated (in blue) and downregulated (in red) proteins are shown.

**Figure 7:**
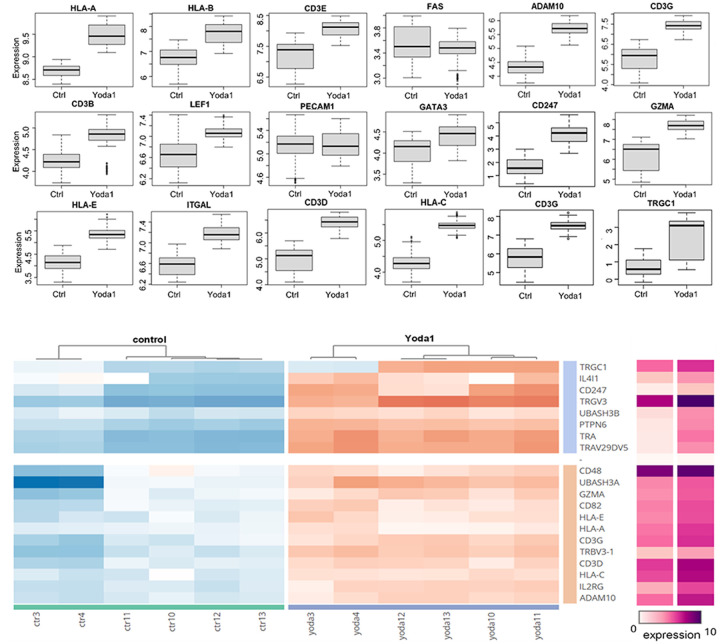
Proteomic analysis showed Yoda1 treatment increased the expression of T cell markers. Clustering and box plots of markers related to immune activation including HLA, TCR and CD3 complexes are shown. Upregulated genes (in orange) and downregulated genes (in blue) in response to Yoda1 treatment are shown. Box plots show key gene expression comparison. The absolute value of each group’s average expression is indicated in purple.

Proteomic analysis also showed the markedly increased expression of the Human Leukocyte Antigen family (HLA) upon Yoda1 treatment. Specifically, HLA-A, HLA-C and HLA-E were upregulated (Fig. 7). These results suggest that upregulation of these HLA molecules along with induction of HIV protein expression (Fig. 2) may impact the recognition and killing of latent cells by cytotoxic T cells and NK cells through class I presentation of HIV antigens.

### Proteomics analysis showed that Yoda1 reactivated latent HIV by downregulation of mRNA processing and ribosome biosynthesis proteins.

**Figure 8:**
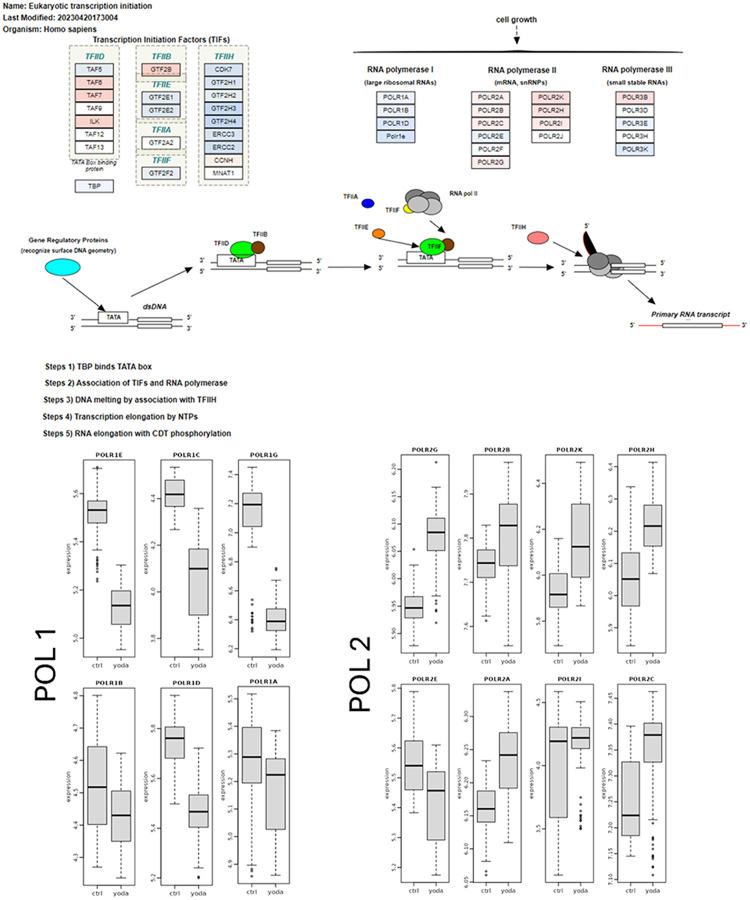
Proteome showed Yoda1-mediated regulation of host RNA polymerase and transcription factors. The polymerase activity diagram is illustrated listing the transcription factors (TF) and the polymerase complexes. Blue (indicates downregulated genes) and red (indicates upregulated genes) highlights represent differential gene expression observed in Yoda1-treated vs control groups. Polymerase 1 subunits and TF2H were dramatically downregulated in response to Yoda1 treatment. In contrast, polymerase 2 complex members were upregulated, although the variations were not statistically significant (Refer to the tables in the supplemental material for the statistical analysis).

Interestingly, we found that Yoda1 regulated both host gene transcription and translation. Regarding transcription, Yoda1 downregulated the components of the RNA polymerase I complex: POLR1G and POLR1E; while upregulating the RNA polymerase II components: POLR1A, POLR1B, and POLR1D (Fig. 8). RNA polymerase 1 decrease was associated with RNA polymerase 2 components increase (POLR2A/B/C/G/K/H and I). While RNA polymerase 1 oversees the production of ribosomal RNAs, RNA polymerase 2 is responsible for the production of messenger RNAs including the replication of HIV that serves as genome for the new viral particles and for the synthesis of viral proteins.

Yoda1 also impacted gene transcription in ACH2 cells by regulating transcription factors (Fig. 8), Specifically Yoda1 either upregulated or downregulated Transcription Initiation Factors (TIFs) depending on the transcriptional regulator (TFIID, B or H).

**Figure 9:**
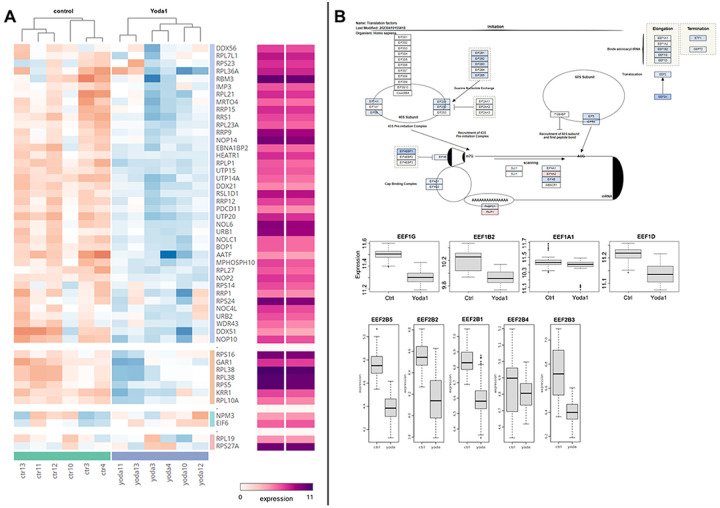
Proteome showed Yoda1 regulated expression of ribosome proteins and translation factors: A) Analysis of nucleolar and ribosomal protein expression showed broad downregulation. Of the 249 proteins clustered, 40 were statistically less abundant in the Yoda1 group. Orange and blue represent respectively the relative up- and down- regulation in the Yoda1 vs control comparison. In purple scale the absolute values of the group average are shown. B) Yoda1 treatment decreased expression of several translation factors. Diagram of the translation process is shown with differential expression of proteins between control and Yoda1 treatment (blue and red denote downregulated and upregulated genes, respectively). Box plots show key elongation factor gene expression comparison.

With respect to gene translational regulation by Yoda1, proteomic analysis revealed downregulation of a set of nucleolar and ribosome factors, that were suggested to affect ribosome function.^36,37^ 49 of 249 of these nucleolar and ribosome factors were detected in the proteomic analysis. 45 of these factors were down regulated in response to Yoda1 treatment with 40 showing a meta.q < 0.05 (Fig. 9A). Although the effects size was modest, the wide impact of Yoda1 treatment on ribosome related proteins suggest the Yoda1 results in an overall decreased ribosomal activity.

Yoda1 also downregulated several structural and functional proteins essential to ribosome activity. Several sub-units of the Eukaryotic Initiation Factor 2b (EIF2B1, EIF2B2, EIF2B5, EIF2B3) were slightly but consistently affected by Yoda1 (Fig. 9). The EEF1 complex, including EEF1A1, EEF1B2, EEF1G and EEF1D were also downregulated in response to Yoda1 (Fig. 9B). These factors are necessary for matching aminoacyl-tRNAs with their corresponding codon on the mRNA during translation. Altogether, these results suggest that the ability of Yoda1 to regulate host and viral gene transcription and translation could aid in the expression of HIV genes and reactivation of latent HIV.

### Transcriptomic analysis revealed the upregulation of T cell markers by Yoda1.

**Figure 10:**
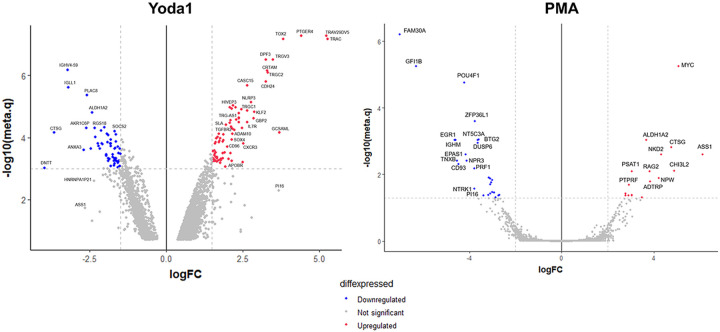
Transcriptomic profile of ACH2 showed induction of distinct T cell activation pathways by Yoda1. Comparison of the transcriptomic profiles of ACH-2 cells treated with Yoda1 and PMA. The two profiles showed different differential expression targets indicating involvement of different pathways. Yoda1-induced latent HIV reactivation was characterized by high expression of TCR receptor members, HIVEP3 and other molecules characteristic of immune activation. Upregulated (in red) versus downregulated (in blue) genes are shown. The logarithm (base 2) of differential expression as fold change (x-axis) versus the negative logarithm base 10 of the meta.q value (y-axis) is shown.

In comparison with our proteomics data, transcriptomic analysis also revealed some interesting results. The transcriptomic profiles of PMA and Yoda1 induction were profoundly different from each other with no overlap among the most up- and downregulated genes (Fig. 10).

**Figure 11:**
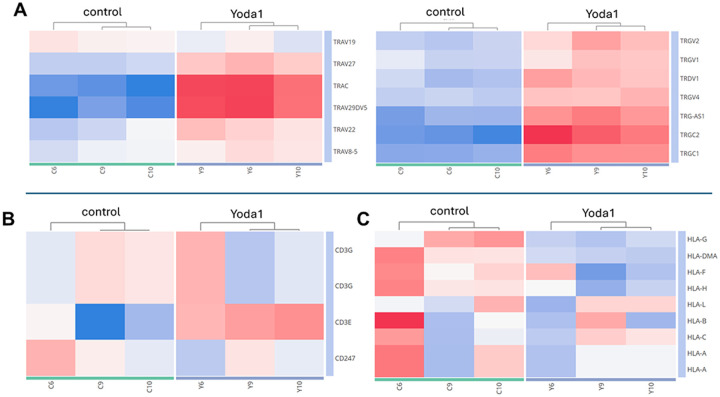
Transcriptomic analysis of Yoda1-mediated upregulation of T cell activation markers. A) Yoda1 had a major effect on the overexpression of a set of TCR receptors belonging to both αβ and γδ T cells. B and C) Transcriptomics analysis of T cell co-receptor CD3 complex and HLA family members. Upregulated genes (in red) and downregulated genes (in blue) in response to Yoda1 treatment are shown.

In comparison with proteomic analysis, transcriptomic analysis also revealed increased expression of T cell receptor complex genes. Among the most up-regulated genes were 5 T cell receptor alpha genes (TRAV27, TRAC, TRAV29DV5, TRAV 22 and TRAV), 7 T cell receptor gamma complex genes (TRGV2, TRGV1, TRGV4, TRG-AS1, TRGC1 and TRGC2) and the T cell receptor delta, TRDV1 gene (Fig. 11A). Regarding CD3 and HLA expression, transcriptomic data showed no significant upregulation of these genes, in contrast to the proteomics analysis (Fig. 11B and C). Altogether, the combined proteomics and transcriptomics data provide a possible explanation for the Yoda1-mediated HIV reactivation via T cell activation mediated by the TCR/CD3 complex.

### Transcriptomics revealed regulation of gene transcription and translation by Yoda1.

**Figure 12:**
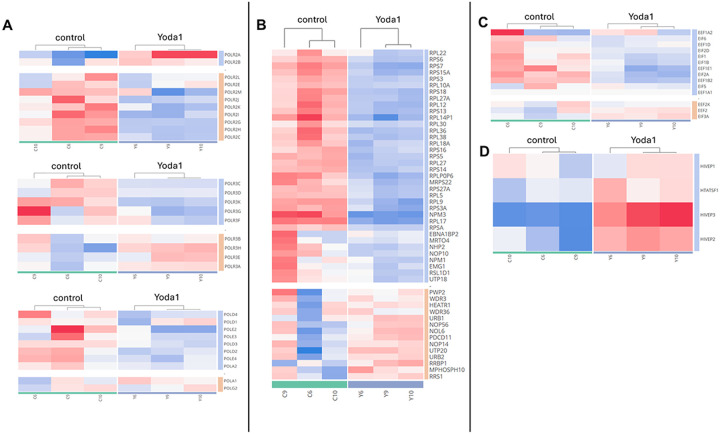
Transcriptomic analysis of Yoda1-mediated regulation of gene transcription and translation factors. A) Yoda1 response correlated with decreased expression of RNA polymerase (POL) 2 components and a variable effect on the expression of the RNA polymerase 3. B) Yoda1 affected ribosome activity by a broad downregulation of genes associated with ribosome biogenesis.^36,37^ C) Yoda1 decreased ribosome activity by down regulating a set of translation factors belonging to the EIF and EEF families. D) Yoda1 increased the expression of two members of the HIVEP family specifically HIVEP2 and HIVEP3 that are involved in Immunoglobulins expression and HIV replication. Upregulated genes (in red) and downregulated genes (in blue) in response to Yoda1 treatment are shown.

Similar to proteomics, transcriptomics showed the effect of Yoda1 on regulating genes associated with the RNA polymerases needed for gene transcription. The mRNAs of the major polymerase 2 subunits (POL2A and POL2B) were upregulated, while the other polymerase 2 subunits (POLR2E/C/E/G/H/I/J/K/L/M) were downregulated. The upregulation of these genes in the proteomic data was observed, but it lacked statistical significance (Fig. 12A).

Transcriptomics, like proteomics, also showed the effects of Yoda1 on ribosome synthesis necessary for protein translation. We performed the analysis of genes relevant for the ribosome biosynthesis suggested by *Jarboui et al*, as performed in the analysis of the proteomic data. Of the 249 genes associated with ribosome synthesis^36,37^,117 were affected by Yoda1 treatment of ACH2 cells. Consistent with proteomic analysis, we found that the majority of the proteins (87 over 117) were downregulated in response to Yoda1, with the downregulation of 36 of these proteins showing statistical significance (meta.q < 0.05) (Fig. 12B). The ribosome genes RPL10A, RPL21, RPL27, RPL38, RPS5, and RPS16 were significantly downregulated in both proteomic and transcriptomic data sets.

In line with the proteomic data, several elongation factors crucial for the protein translation were downregulated by Yoda1 with the exception for EEF2, EEF2K and EIF3A (Fig. 12C). Taken together, the transcriptome suggests the ability of Yoda1 to regulate both host transcription and translation likely promotes HIV gene expression and latent HIV reactivation.

### Transcriptomic analysis showed that Yoda1 upregulated the HIVEP transcription factors involved in T cell activation and HIV gene expression.

Transcription factors such as NF-kB play a key role in T cell activation as well as in HIV LTR transactivation and gene expression.^38–40^ Transcriptomic analysis showed that Hivpep2 and 3 (known as Schnurri) expression was increased by Yoda1 (Fig. 12D). HIVEP2 and 3 are zinc finger transcription factors that bind to the recombination signal sequence (Rss) flanking the V, D, and J gene segments of the immunoglobulin gene and enhance HIV transcription via LTR transactivation.^41,42^ Taken together, these results revealed the possible involvement of T cell activation in latent HIV reactivation and reversal of HIV latency mediated by Yoda1 through the regulation of transcription factors involved in T cell activation, HIV LTR transactivation and gene expression.

## DISCUSSION

Despite the ability of ART to suppress HIV in PWH, there is no cure for HIV due to the remaining presence of latent viral reservoirs that are unperturbed even by the most powerful antiretroviral drugs available. The goal of kick-and-kill HIV cure strategy is to reactivate latent HIV and destroy these latent reservoirs, resulting in the complete elimination of the virus from the host. LRAs are being developed to reactivate latent HIV. Unfortunately, LRAs previously tested have failed in clinical trials likely due to inefficient reactivation coupled with the poor clearance of latent HIV-infected cells.^17,18,20,22,32,43,44^ Therefore, it is paramount and a research priority to develop more effective kick-and-kill strategies including the development of new LRAs that can reactivate latent HIV more effectively to ultimately achieve HIV cure.^45^

Our study investigated Yoda1, a synthetic Piezo1 agonist, as a novel LRA. We demonstrated that Yoda1 can effectively reactivate latent HIV in the ACH2 T cell line. The Yoda1-induced latent HIV reactivation was mediated by Piezo1. Yoda1 induced the production of infectious HIV and increased HIV gene expression in ACH2 cells. Latent HIV reactivation by Yoda1 was associated with T-cell activation, HLA upregulation and the modulation of host and viral transcription and translation that favors viral gene expression.

The ability of Piezo1 to reactivate HIV is not surprising given that prior studies showed that in CD4 T cells, Piezo1 is involved in T cell activation via calpain activation and organization of cortical actin scaffold, as well as Treg development requiring TGFβ and integrin-dependent chemotactic migration.^29,30^ Interestingly, however, the signaling pathways induced by Yoda1 in ACH2 cells are novel and distinct from prior studies on LRAs and Piezo1-mediated T cell activation. This could likely be due to the presence of latent HIV in ACH2 as well as in the diversity of the HIV-infected CD4 T cell reservoir. We plan future studies involving Yoda1 treatment of uninfected CD4 T cells in the absence of latent HIV to assess how latent HIV infection influences Yoda1-mediated T cell activation.

Integrated proteomic and transcriptomic analyses revealed a novel HIV reactivation profile in latent cells treated with Yoda1. Proteomic analysis identified 485 differentially expressed proteins post-Yoda1 treatment, with 411 upregulated and 74 downregulated proteins. Upregulated proteins were primarily involved in immune response pathways, including TCR and the co-receptor CD3 complex, as well as the HLA molecules, which play critical role in antigen presentation, T cell recognition and activation.

Notably from our Omics analyses, Yoda1 increased the expression of TCR co-receptor complex components such as CD3D, CD3E, CD3G, and CD247, crucial for T cell signaling, were increased upon reactivation of latent HIV in ACH2 cells. HIV latency reversal was found to be inefficiently induced by T cell receptor (TCR) engagement, perhaps due to a TCR engagement deficit, while agents that bypass proximal TCR signaling can induce downstream signals.^46^ Interestingly, like PMA, anti-CD3/CD28 is also a potent LRA *in vitro*, better than clinically tested LRAs.^47^ These findings suggest that Yoda1 may serve as an effective LRA by upregulating TCR/CD3 complex, including TCR and CD3, creating a more effective TCR engagement necessary for latent HIV reactivation or reversal of latency.

Upregulation of HIV gene expression is likely due to the Yoda1-mediated LTR transactivation and T cell activation involving NF-κB and HIVEP2 and 3.^38,40,42,48^ Previously, the activation of T cells and HIV transcription were found to share common upstream signaling pathways, including reliance on the transcription factor NF-κB.^39^ It is noteworthy that Yoda1 increased the expression of transcription factors HIVEP2 and 3, which in concert with NF-κB could not only augment T cell activation but also HIV gene expression through LTR transactivation. As an LRA, Yoda1 could reactivate latent HIV through T cell activation and LTR transactivation involving transcriptional regulators such as HIVEP2 and 3.

Yoda1 also upregulated HLA molecules including HLA-A, HLA-C, and HLA-E, enhancing antigen presentation and immune surveillance. Upregulation of HLA molecules along with HIV protein in reactivated latent cells could render these cells susceptible to CTL killing. HLA-A bound to HIV peptides could lead to the killing by Cytotoxic T cells; while HLA-C bound to HIV peptides could mediate recognition by both natural killer and T cells.^10,44,49^ Upregulation of HLA-E could lead to the binding of NK cells and a small subset of T cells in the peripheral blood via CD94/NKGK2A/B/C NK cell receptors. Engaging CD94/NKG2A or CD94/NKG2B induces an inhibitory effect on the cytotoxic activity of the NK cell to prevent cell lysis.^50^ Dichotomously however, binding of HLA-E to CD94/NKG2C (KLRC2) triggers expansion of NK cell subsets in antiviral responses.^51^ The precise role of these HLA molecules in the elimination of latent reservoirs needs to be further investigated.

In addition to immunomodulation, Omics analyses demonstrated that Yoda1 impacts ribosome and nucleolar function. Proteomic analysis showed that Yoda1 downregulated several proteins associated with ribosome biosynthesis and nucleolar activity, including proteins involved in ribosomal RNA (rRNA) processing and structural components of the ribosome. This downregulation results in a decrease overall ribosomal activity, which may have implications for cellular protein synthesis and viral replication. Transcriptomic analysis further confirmed that Yoda1 induced changes in gene expression related to ribosome function characterized by a modest but wide downregulation of nucleolar and ribosomal components fundamental for the ribosome biogenesis.

Omics analyses also revealed that Yoda1 treatment impacted RNA polymerase complexes, specifically decreasing components of RNA polymerase I, which is responsible for rRNA synthesis while increasing components of RNA polymerase II, which is responsible for mRNA synthesis. This shift suggests that Yoda1 may promote viral replication by favoring the transcription of HIV mRNA over the synthesis of rRNA, altering the transcriptional machinery in favor of viral gene expression. An important consequence is triggering host defense in that inducing HIV expression on latent infected cells could lead to the destruction of the reservoir. Increased expression of envelope protein on the cell surface could trigger antibody dependent cellular cytotoxicity.^52,53^ Furthermore, increased HIV protein expression could lead to enhanced CTL cell killing of latent cells via HLA presentation of HIV peptides and by the recognition of antibody-HIV envelop complex by CD16 receptor of the NK cells.

It is worth noting that proteomic and transcriptomic data showed some overlap. There were differences between the two analyses, however. For example, proteomic but not transcriptomic analysis was able to detect statistically significant upregulation of members of the CD3/TCR complex and HLA family. Differences between proteomic and transcriptomic analyses are commonly observed. In fact, assay detection sensitivity and specificity as well as the presence of post-translational modifications, RNA and protein stability were found to account for Omics analysis differences.^54,55^ This highlights the complexity of cellular responses to Yoda1 and underscores the importance of multi-omics approach to fully delineate the role of Yoda1 in latent HIV reactivation and latency reversal.

**Figure 13:**
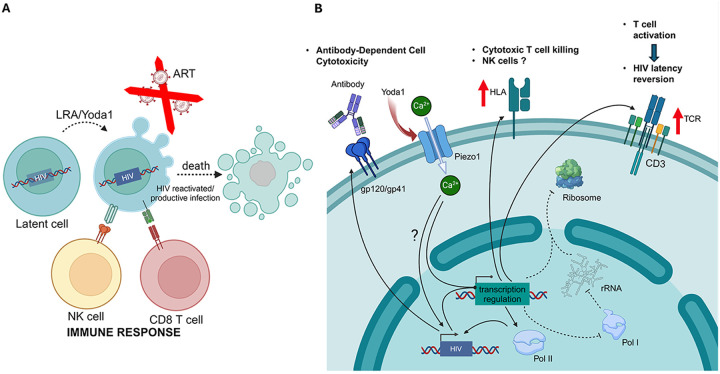
Proposed model for Yoda1 as a latency-reversing agent for HIV cure. A) Representation of the Kick-and-Kill Theoretical Framework: Achieving a sterilizing cure for HIV requires the complete elimination of the HIV reservoir responsible for re-establishing infection shortly after antiretroviral therapy (ART) is discontinued. HIV in its latent state is characterized by the absence or low level of viral gene expression, enabling the virus to become undetectable to both the immune system and drug targeting. The kick-and-kill approach relies on developing Latency Reversal Agents (LRAs), which are compounds designed to reactivate viral replication. Once reactivated, the newly synthesized virus can be targeted by co-administered ART, preventing it from infecting new cells. Latent cells that transition to an active state will undergo cell death through either the host immune response (including NK and CD8 T cells) or the virus’s cytopathic effect. HIV reactivation has been associated with the expression of Death Receptors (DR), which, upon interaction with TRAIL on the NK cell surface, trigger cell death. Additionally, NK cells can recognize opsonized HIV proteins on the cell surface via CD16 leading to the killing of infected cells. **B) Proposed mechanism of HIV reactivation and killing of latent cells mediated by Yoda1:** Based on our preliminary data, we proposed a model on how Yoda1 acts as an LRA to reactivate HIV and render latent cells susceptible to immune-mediated killing. Yoda1 is a selective chemical agonist of the mechanosensor Piezo1. Upon activation by Yoda1, Piezo1 acts as a calcium channel, leading to cellular calcium uptake and triggering a series of transcriptional effects, including the reactivation of the latent HIV genome. The mediators of HIV reactivation are the subject of future investigation. The cascade of cellular events initiated by Yoda1 results in the modulation of RNA synthesis, driven by the upregulation of RNA polymerase II and the downregulation of RNA polymerase I, which is involved in the synthesis of ribosomal RNA. Yoda1 impairs the ribosome function through the downregulation of a set of proteins necessary for the proper function of this organelle. Additionally, Yoda1 induces the overexpression of several genes related to the immunological role of T cells, including an increase in all components of the T cell receptor (TCR) and CD3. This TCR/CD3 overexpression sensitizes T cells to activation, an event associated with HIV latency reversion. Yoda1 also increases expression of HLA (human leukocyte antigen) proteins, such as HLA-A, HLA-C, and HLA-E proteins. In addition, Yoda1 induces LTR transactivation leading to the production of HIV. This increase in HIV protein production along with increased expression of HLA proteins is promising, as it may facilitate the destruction of latent cells by class I presentation of HIV peptides to activate cytotoxic T cells, NK cells and antibody-dependent cellular cytotoxicity (ADCC).

Based on our collective findings, we propose a model for how Piezo1 reactivates latent HIV or reverses latency and contributes to the elimination of latent HIV reservoir (Fig. 13A and B). Briefly, Yoda1 interacts with Piezo1, leading to cellular calcium uptake and triggering a series of transcriptional effects, including the reactivation of the latent HIV genome. The mediators of HIV reactivation are the subject of future investigation. The cascade of events initiated by Yoda1 results in the modulation of RNA synthesis, driven by the upregulation of RNA polymerase II and the downregulation of RNA polymerase I, which is involved in the synthesis of ribosomal RNA. Yoda1’s influence on the ribosome extends to the downregulation of a set of proteins necessary for the proper function of this organelle.

Additionally, Yoda1 induces the over expression of several genes related to the immunological role of T cells, including a significant increase in all components of the T cell receptor (TCR) and CD3. This TCR/CD3 over expression sensitizes T cells to activation, an event associated with HIV latency reversal. Several members of the HLA family, including HLA-A, HLA-C, and HLA-E are also upregulated by Yoda1. Furthermore, Yoda1 upregulates HIV gene expression through LTR trans activation. This increased expression of HLAs as well as increase HIV protein expression facilitates the clearance of latent cells by class I presentation of HIV peptides activating the cytotoxic CD8 T cells or antibody-depending cellular cytotoxicity.

There are possibly other host factors or mechanisms that play a role in Yoda1-mediated latent HIV reactivation. Based on previous studies on the role of calcium influx in T cell activation, citrullination might be involved in HIV reactivation in that the calcium influx triggered by Piezo1 activation through Yoda1 stimulation leads to histone citrullination that is mediated by calcium-dependent Peptidyl Arginine Deaminases (PADs).^56^ In support of this hypothesis, PAD2 activation was associated with Th17 cell activation, and treatment with Yoda1—a PIEZO1 activator—was linked to increased histone H3 citrullination.^33,56^ These observations suggest that Yoda1 could be a potent LRA because it possibly activates T cells through histone citrullination mediated by PAD4.^57^

To deepen our understanding of the effect of Yoda1 on T cells, we plan to perform post translational analysis to determine the phosphorylation cascade associated with the T cell markers and receptor signaling upregulation.^58^ The study of protein phosphorylation through phosphoproteomics would be a key analysis for understanding the signaling landscape in the response of latent cell reservoir to Yoda1 stimulation. The phosphorylation of intermediate proteins is a common feature that is integral to a variety of cell-signaling cascades mediated by the CD3/TCR complex, NF-kB and several others.^58,59^

Yoda1 as a new LRA is innovative. Unfortunately, to date, candidate LRAs have failed to eliminate or substantially reduce the latent reservoir.^18–20,32,60^ Yoda1 serves as a method for reactivating a reservoir of latent HIV-1 in PWH. Conceptually, the identification of novel pathways for HIV reactivation in T cells and other cell reservoirs by Yoda1 is innovative. Yoda1 stimulates Piezo1 activation pathways unique from prior LRAs (Fig. 13), which can be leveraged to tweak Yoda1/Piezo1 agonists and develop new LRAs and therapeutic strategies to efficiently reactivate latent HIV. Other mechanosensors (i.e. Piezo2) may have reactivation potential in latent cells that can be easily tested.^61^

Piezo1 is expressed broadly in many cell types, including macrophages, which are reservoirs for latent HIV.^8,9,11,17^ Research indicates that non-T cells can harbor HIV, and the Yoda1 agonist may effectively reactivate these other latent HIV-infected non-T cells.^8,11^ Use of Yoda1 or a more effective Piezo1 agonist (Yoda2, KC159, Jedi1 or Jedi2^62,63^) in combination with other safe LRAs is a possible route to efficiently reactivate latent cells. Current LRAs activate certain pathways such as histone acetylation (Vorinostat, Panobinostat or Romidepsin) that are different or may overlap with Yoda1-Piezo1 signaling pathways and altogether may be synergistic at reactivating HIV.^19,21,44^

Administering Yoda1 with a therapeutically effective amount of an antiretroviral composition can be a more efficient treatment. It may prevent the establishment of latent HIV if administered immediately after exposure to the virus. Various “kill” strategies including enhanced killing by CTLs, NK cells and antibodies (i.e. ADCC) are being developed, and these can be combined with Yoda1.^49,52,64,65^

Yoda1 has a good safety profile, and it is easily produced. Yoda1 has been proven to have safety and good tolerance in vitro and in mouse models. Mice continuously treated with Yoda1 at doses as high as 213 ug/kg for 35 days did not develop any significant health issues or notable changes in their blood biochemistry.^66^ Yoda1 is readily available and manufactured for clinical testing and eventual use as an LRA in PWH.

Future research should focus on validating these findings in animal models of latent HIV as well as in the clinical setting. These studies should evaluate the efficacy of Yoda1 in reactivation of latent cells and reducing latent reservoir in primary T cells, macrophages and other cells from a larger cohort of PWH. In vivo testing in humanized mouse and non-human primate models to assess the viability of Yoda1 as therapeutic candidate should be conducted. Integrating single-cell omics techniques using PWH patient samples will be needed to investigate host variability in latent reservoir responses to Piezo1 activation, mainly due to reservoir population heterogeneity and reservoir size differences. These studies will be crucial in our understanding of the complexity of latent HIV infection and reactivation, as well as the optimization of Piezo1-based HIV cure strategies.

## Data Availability

The data sets used and analyzed in this study are available from the corresponding authors (mcayabya@nova.edu and abontemp@nova.edu) upon request.
